# Endoscopic Ultrasonography in the Diagnosis and Treatment Strategy Choice of Esophageal Leiomyoma

**DOI:** 10.6061/clinics/2017(04)01

**Published:** 2017-04

**Authors:** Ling-Jia Sun, Xin Chen, Yi-Ning Dai, Cheng-Fu Xu, Feng Ji, Li-Hua Chen, Hong-Tan Chen, Chun-Xiao Chen

**Affiliations:** I Department of Gastroenterology, The First Affiliated Hospital, Zhejiang University, Zhejiang, China; II Department of Gastroenterology, Ningbo No. 2 Hospital, Zhejiang, China

**Keywords:** Endoscopic Ultrasonography, Esophageal Leiomyoma, Diagnosis and Treatment Strategy

## Abstract

**OBJECTIVES:**

Esophageal leiomyoma is the most common benign tumor of the esophagus, and it originates from mesenchymal tissue. This study analyzed the clinicopathological characteristics of esophageal leiomyoma and aimed to evaluate the role of endoscopic ultrasonography in the diagnosis and treatment selection for these lesions.

**METHODS:**

Two hundred and twenty-five patients who had suspected esophageal leiomyomas in endoscopic ultrasonography were enrolled at the Endoscopy Center of The First Affiliated Hospital, Zhejiang University from January 1^st^, 2009 to May 31^th^, 2015. The main outcomes included the demographic and morphological characteristics, symptoms, comparisons of diagnosis and treatment methods, adverse events, and prognosis.

**RESULTS:**

One hundred and sixty-seven patients were diagnosed as having an esophageal leiomyoma by pathological examination. The mean patient age was 50.57±9.983 years. In total, 62.9% of the lesions originated from the muscularis mucosa, and the others originated from the muscularis propria. The median distance to the incisors was 30±12 cm. The median diameter was 0.72±0.99 cm. As determined by endoscopic ultrasonography, most existing leiomyomas were homogeneous, endophytic, and spherical. The leiomyomas from the muscularis mucosa were smaller than those from the muscularis propria and much closer to the incisors (*p*<0.05). SMA (smooth muscle antibody) (97.2%) and desmin (94.5%) were positive in the majority of patients. In terms of treatments, patients preferred endoscopic therapies, which led to less adverse events (e.g., intraoperative bleeding, local infection, pleural effusion) than surgical operations (*p*<0.05). The superficial leiomyomas presented less adverse events and better recovery (*p*<0.05) than deep leiomyomas.

**CONCLUSION:**

Endoscopic ultrasonography has demonstrated high accuracy in the diagnosis of esophageal leiomyomas and provides great support in selecting treatments; however, EUS cannot completely avoid misdiagnosis, so combining it with other examinations may be a good strategy to solve this problem.

## INTRODUCTION

Esophageal leiomyoma is a common type of esophageal tumor. Although the morbidity varies across different studies, it is undoubtedly the most common benign tumor of esophagus, accounting for approximately 70% of esophageal submucosal tumors 1-4. It is considered an irreversible lesion, and it originates from mesenchymal tissue. Most cases occur in adults aged 20 to 80 years, with the average age range of 40 to 50 years 1,5,6. The patients often have diverse symptoms, and the symptoms and their severity level seem to be related to lesion location and shape 7,8. Because of the development of endoscopic technology, many non-symptomatic patients with esophageal leiomyomas are diagnosed during normal physical check-ups.

Esophageal leiomyomas always occur in deep layers of the esophagus 9. Tissue biopsy by forceps may present false-negatives. The conventional endoscopy could produce misdiagnoses because of differences in personal experiences. Endoscopic ultrasonography (EUS) has the ability to check lesions as well as the adjacent organs and structures 10. It can easily evaluate the depth of lesions, growth directions, metastasis and other useful parameters.

There are many therapies that are currently available to treat with esophageal leiomyomas. Doctors used to perform a thoracotomy to enucleate the whole lesion 11. By the 1990s, thoracoscopy and laparoscopy were widely applied in this field 12-14. After that, endoscopy and endoscopic operation were developed and widely used. Several treatments have been deployed on esophageal leiomyomas and have achieved good results.

The purpose of this study was to summarize the clinicopathological characteristics of esophageal leiomyoma and to analyze the significance of EUS on the diagnosis and treatment strategy choice of esophageal leiomyomas.

## METHODS

### Subjects

The patients who were enrolled in this study were those suspected to have an esophageal leiomyoma at the Endoscopy Center of The First Affiliated Hospital, Zhejiang University from January 1^st^, 2009 to May 31^th^, 2015. The total number was 225. The study protocol was approved by the Ethics Committee of The First Affiliated Hospital, Zhejiang University. All patients involved in this study were informed of the potential hazards and signed written informed consent statements.

### EUS procedures

The EUS device was an ultrasound gastroscope (Olympus, EUS2000) with a 12 MHz miniprobe (Olympus, UM-2R). EUS examinations utilized the same preparations as conventional gastroscopy.

The suspected esophageal leiomyoma diagnosis criterion was a homogeneous and hypoechoic structure with clear borders in the ultrasound image and a submucosal lesion in the endoscopic image, with no destruction of the mucosa.

### Statistical analysis

The parameters that we recorded included the demographic information of each patient, morphological characteristics of the lesions by EUS and CT scan, pathological and immunohistochemical staining features of the specimens, treatments and follow-up conditions. The immunohistochemical staining markers mainly consisted of desmin, CD117, SMA, S100, CD34 and DOG1.

The demographics and clinicopathological parameters were analyzed statistically by IBM SPSS statistics version 19.0 software. Statistical analyses included the Mann-Whitney U test and the χ^2^ test. The statistical significance was defined as *p*<0.05.

### Follow-up

This study was a retrospective study. The follow-up methods included telephone calls and repeated patient visits to the hospital. The follow-up period was calculated from the date that the EUS was implemented.

## RESULTS

### Characteristics of study subjects

A total of 225 patients were diagnosed as either having an esophageal leiomyoma or a suspected leiomyoma under EUS. Among them, 182 patients gave pathological specimens from endoscopic treatments, surgery and biopsy. The lesions of 167 patients were confirmed to be consistent with the preoperative diagnosis by pathological examination (91.75%). Fifteen misdiagnosed lesions were actually a mesenchymoma, inflammatory mass, schwannoma or esophageal carcinoma.

The esophageal leiomyoma patients included 100 males (59.9%) and 67 females (40.1%). The mean age of these patients was 50.57±9.983 years (range: 23-72 years), including 51.46±10.434 years for male patients and 49.24±9.339 years for females (Table 1).

The median length of the total hospitalization period was 8±6 d, and the median hospitalization time after treatments was 4±3 d.

### Clinicopathological characteristics of esophageal leiomyoma

#### Endoscopic characteristics of EUS

Five cases out of the 167 patients had multiple lesions. Four patients had two lesions, and the other one had three.

The lesions of 105 patients originated in the muscularis mucosa (62.9%). Of these, 37.1% infiltrated the muscularis propria (62/167). Of the lesions that infiltrated the muscularis propria, 14.5% infiltrated the superficial muscularis propria, and 85.5%, the non-superficial layer.

In terms of anatomic locations, the median distance to the incisors was 30±12 cm (18-42 cm). The percentages of lesions located in the upper, middle, and lower segments of esophagus were 16.8%, 41.3% and 41.9%, respectively.

In regard to lesion size, the median diameter of all esophageal leiomyomas was 0.72±0.99 cm (0.2-8.5 cm). The diameter of leiomyomas localized in the muscularis mucosa and muscularis propria were 0.52±0.34 cm and 2.07±1.22 cm, respectively.

During EUS observation, 86.8% of the leiomyomas were determined to be homogeneous echo (145/167). The leiomyoma morphologies included spherical (79.6%), fusiform (9.6%), polypoidal (0.6%), and even 10.2% irregular types. Ten patients had lesions that were exophytic type (6.0%).

When comparing the lesions from the muscularis mucosa and muscularis propria, there were no significant difference related to gender (*p*=0.540) or symptoms (*p*=0.949). More irregular- and exophytic-type leiomyomas originated in the muscularis propria than in the muscularis mucosa (*p*<0.05). More muscularis propria lesions had echoes that were homogeneous (*p*<0.05).

Whether EUS could accurately diagnose the esophageal leiomyoma was relative to many parameters of the lesion, including shape, growth direction, echo, size, origination and location (Table 2).

#### Symptoms

Forty-eight of the 167 patients were diagnosed unexpectedly during medical checkups. Among the 119 patients with symptoms, the most common one was upper abdominal discomfort (40.1%). Approximately 9.0% of the patients suffered from acid reflux and heartburn (15/119). Dysphagia also occurred in 9.0% (15/119) of the patients. Other symptoms included thoracalgia (6.6%), dyspepsia (4.2%), paraesthesia pharynges (1.8%) and nausea (0.6%).

Neither the demographic nor endoscopic characteristics of the leiomyoma had an effect on symptom occurrence; however, specific symptoms, such as dysphagia as a classic symptom, presented statistical correlation with the distance between incisors and lesions.

#### Comparison with CT scan

Among esophageal leiomyoma patients, 83 patients were given CT scans for assistance. Twenty-nine patients were reported as having normal esophagi (34.9%). Masses were found in 54 patients, of which 4 patients were diagnosed as having esophageal leiomyomas (4.8%), and 10 patients had ambiguous outcomes (12.0%).

When compared with the EUS observations, esophageal leiomyomas from muscularis propria, with large diameters or inhomogeneous echoes, were easier to detect by CT scan (Table 3).

#### Immunohistochemical staining

Only 75 patients agreed to an immunohistochemical examination but not every patient's sample displayed all of the biomarkers. SMA and desmin were positive in 97.2% and 94.5% of the samples, respectively, and these were the only markers that were observed in over 90% of the samples. The specific information is detailed in Table 4.

#### Treatments

A total of 166 patients were under treatments in our study. Various therapy methods have been used to treat esophageal leiomyomas. With the exception of the 23 patients who received surgical operations (13.8%), the majority of the patients preferred endoscopic treatments. Specifically, most patients chose endoscopic trepanned resection (ETR) (37.7%). Thirty patients received submucosal tunneling endoscopic resection (STER) (18.56%). The numbers of patients who underwent endoscopic mucosal resection (EMR), endoscopic submucosal dissection (ESD) and endoscopic submucosal excavation (ESE) were 28, 11 and 10, respectively. Electric cauterization was implemented on the remaining one patient.

The intraoperative complications mainly included perforation and intraoperative bleeding (>50 ml). Perforation occurred in 7 patients (4.2%), and intraoperative bleeding, in 18 patients (10.8%). Among the 143 patients who had endoscopic operations, only two patients endured intraoperative bleeding >50 ml. The operation methods had no effect on the occurrence of perforation (*p*=1.00). Endoscopic operations led to less intraoperative bleeding than surgical operations (*p*<0.05) (Table 5).

In comparing leiomyomas from the muscularis mucosa and muscularis propria, there were many differences between these two types of lesions (Table 6). The originating location might significantly impact the therapy choices and prognosis.

Various treatments were used depending on the different originating depths. ESD and EMR were often performed on leiomyomas from the muscularis mucosa. For leiomyomas from the muscularis propria, the main therapies were surgical operations and endoscopic therapies, including ESE and STER. Elderly patients with small, homogeneous, regular type lesions preferred endoscopic therapies more than surgical operations (Table 7).

#### Prognosis

The follow-up methods mainly included repeated patient visits to the hospital and telephone calls. A total of 124 patients had subsequent visits after hospital discharge. The follow-up rate was 74.7%. Among these individuals, three had endoscopic ultrasonography, four had CT scans, and the others chose electronic gastroscopy for examination. The mean follow-up time was 26.7±17.6 months.

The postoperative adverse events were divided into short-term and long-term. The short-term postoperative adverse events that we recorded were delayed bleeding, local infection, pneumomediastinum, pneumothorax and pleural effusion. The most common one was pleural effusion, which happened in 28 patients (16.9%). The 23 patients who had surgical operations all suffered from pleural effusion. The incidences of delayed bleeding, local infection, pneumomediastinum, and pneumothorax were 1.2%, 5.4%, 3.6% and 3.0%, respectively. Of 166 patients in our study, 33 had these events. Local infection and pleural effusion occurred more commonly after surgical treatments. In terms of short-term postoperative adverse events, endoscopic treatments might be more advantageous than surgical methods (Table 8).

The long-term adverse events included recurrence, cicatrization, esophageal stenosis, diverticulum and residual lesions. Thirty-six patients formed scar tissue in the esophagus (21.7%). Three patients had residual lesions. Only one patient developed esophageal diverticulum. There was no significant difference between endoscopic and surgical treatments in terms of long-term adverse effects. Lesions originating from the muscularis propria exhibited the same long-term effects after ESE or STER as with surgical treatments.

## DISCUSSION

With the use of EUS, our study reveals the peak incidence age of esophageal leiomyoma is approximately 40-60 years. Male patients are slightly more likely to develop these lesions than females 1,15. It has been established that 83.2% of these lesions are located in the middle, lower segment of the esophagus, and the percentage of symptomatic individuals is 71.3%. Dysphagia occurs more frequently in patients with lower lesions. Furthermore, none of the symptoms have any correlation with lesion location. Compared with previous studies, the percentage of symptomatic individuals is obviously higher 7,16. This result may be because of an admission bias, the rising attention to self-health, nerve sensitivity and mental fluctuation.

In the past, conventional endoscopy and CT scans were often used to detect esophageal lesions. More than half of the lesions grow from the muscularis mucosa. Conventional endoscopy can easily detect superficial lesions, but it cannot evaluate origination, growth direction and some other characteristics. In addition, approximately 40% of esophageal leiomyomas originate from the muscularis propria, which may increase the difficulty of diagnosis and biopsy. All these situations make it very hard to differentiate esophageal leiomyomas from other analogous lesions.

In our research, CT scans are good at discovering large, irregular leiomyomas with deep origination. Depending on its sharpness, it may not be easy to clearly identify the nature of the lesions.

EUS has significant advantages over these two methods. It can discover lesions, and assess depth, size, shape, and growth direction. Finally, it can distinguish the nature of lesions. It can significantly impact the diagnosis and selection of the treatment strategy. EUS had good sensitivity in our study. Some esophageal leiomyomas with specific characteristics are diagnosed more easily, including the endophytic type, inhomogeneous echo, and superficial origination. Although this may be related to the doctor's experience, it also reflects the endoscopic characteristics. Based on this information, combining conventional endoscopy and CT scan for screening, followed by EUS could allow for lesions to be observed more precisely.

Esophageal leiomyomas from the muscularis mucosa tend to have a better prognosis than the ones from the muscularis propria. After endoscopic treatment, the patients with superficial lesions can resume their diets and leave the hospital earlier. They suffer less postoperation adverse events. The possible reasons for this difference may include smaller sizes, more regular shapes, and growth toward the lumen.

Currently, most patients choose endoscopic therapy to treat superficial esophageal leiomyomas. When compared with surgical operation, it reduces the chance of intraoperative hemorrhage and short-term adverse events after treatments. It appears to be much safer. In terms of leiomyomas from the muscularis propria, there are also some good endoscopic methods that can be used, such as ESE and STER 17. They can achieve similar curative effects and prognosis as surgical treatments. EUS is not only good for diagnosing esophageal leiomyoma but also for contributing to treatment strategy choice.

On the other hand, EUS cannot completely avoid misdiagnoses, especially for some tumors in mesenchymal tissue. In our study, misdiagnosed lesions may contain mesenchymomas and schwannomas. Combining EUS with other examinations may be a good method to solve this problem. It will be a effective way, if the diagnoses combined with histopathology. These three lesions share some commonalities when observed during EUS and have similarities in microscopic morphology. The definite diagnosis is assisted by immunohistochemical staining. Leiomyomas are usually positive for desmin, SMA, CD117, CD34 and DOG-1 at low levels. Schwannomas have typical S100 positivity. Mesenchymomas are positive for CD117, CD34 and DOG-1 at a high level. Several types of biopsies can be implemented by EUS. Microscopic examinations and immunohistochemical staining have the ability to distinguish the nature of lesions.

In conclusion, our research reveals the recent clinicopathologic developments of esophageal leiomyoma. EUS exhibits high accuracy in esophageal leiomyoma diagnosis and provides great support in selecting treatments.

## AUTHOR CONTRIBUTIONS

Sun LJ wrote the manuscript. Chen X, Dai YN, Xu CF, Ji F, Chen LH, Chen HT collected and analyzed the data. Chen CX designed the study.

## Figures and Tables

**Table 1:** Patients’ basic characteristics.

Age (years) [mean±SD (range)]	50.57±9.983 (23-72)
Male (N)	100
Female (N)	67
Length of total hospitalization period (days) [median±QR]	8±6
Hospitalization time after treatments (days) [median±QR]	4±3
muscularis mucosa (N)	105
muscularis propria (N)	62
Distance to incisors (cm) [median±QR (range)]	30±12 (18-42)
Location	
Upper (N)	28
Middle (N)	69
Lower (N)	70
Diameter [median±QR (range)]	0.72±0.99 (0.2-8.5)

**Table 2:** EUS characteristics of confirmed and suspected leiomyomas.

		EUS confirmed	EUS suspected	*p* value
shape (N)	irregular type	11	6	*p*<0.05
	other types	144	6	
growth direction (N)	endophytic type	148	9	*p*=0.02<0.05
	exophytic type	7	3	
echo (N)	homogeneous	143	2	*p*<0.05
	heterogeneous	12	10	
origination (N)	muscularis mucosa	105	0	*p*<0.05
	muscularis propria	50	12	
distance to incisors (cm)		29±11	39±9	*p*<0.05
lesion diameter (cm)		0.65±0.74	3.40±2.18	*p*<0.05

**Table 3:** EUS characteristics of leiomyomas in comparison with CT scans.

	CT missing	CT discovery	*p* value
diameter (cm)	0.56±0.58	1.87±2.29	*p*<0.05
muscularis mucosa (N)	19	20	*p*=0.01<0.05
muscularis propria (N)	10	34	
irregular type (N)	27	39	*p*=0.03<0.05
other types (N)	2	15	
endophytic type (N)	28	45	*p*=0.16
exophytic type (N)	1	9	

**Table 4:** Immunohistochemical staining of the leiomyomas.

	Positive	N	Percentage (%)
Desmin	69	73	94.5
CD117	20	74	27.0
SMA	70	72	97.2
S100	1	66	1.5
CD34	27	70	38.6
DOG1	4	43	9.3

**Table 5:** Intraoperative complications of endoscopic operations.

Complication		endoscopic operation	surgical operation	*P* value
perforation (N)	Y	6	1	*p*=1.00
	N	137	22	
intraoperative bleeding (N)	Y	2	16	*p*<0.05
	N	141	7	

**Table 6:** Characteristics of esophageal leiomyomas from the muscularis mucosa and muscularis propria.

	muscularis mucosa	muscularis propria	*p* value
no symptoms (N)	75	44	*p*=0.95
age	51.90±9.38	48.31±10.64	*p*=0.02<0.05
distance to incisors (cm)	27.00±10.00	35.50±11.00	*p*<0.05
lesion diameter (cm)	0.52±0.34	1.87±1.58	*p*<0.05
irregular type (N)	0	17	*p*<0.05
exophytic growth (N)	1	9	*p*<0.05
homogeneous echo (N)	2	20	*p*<0.05
EUS confirmed (N)	105	50	*p*<0.05
intraoperative bleeding>50 ml (N)	1	17	*p*<0.05
intraoperative perforation (N)	0	7	*p*<0.05
hospitalization time after treatment (days)	3±2	6±2	*p*<0.05
open diet time (days)	1±1	4±3	*p*<0.05

**Table 7:** Characteristics of patients who underwent ESE+STER and surgical operation.

	ESE+STER	surgical operation	*p* value
male	25	14	*p*=0.93
female	15	8	
age	50.30±10.50	44.68±10.13	*p*=0.046<0.050
symptom	31	13	*p*=0.13
no symptom	9	9	
EUS confirmed	35	15	*p*=0.13
EUS suspected	5	7	
irregular type	35	10	*p*<0.05
other types	5	12	
endophytic growth	37	16	*p*=0.08
exophytic growth	3	6	
homogeneous echo	33	9	*p*<0.050
inhomogeneous echo	7	17	
distance to incisors (cm)	36.00±11.00	35.00±11.25	*p*=0.70
lesion diameter (cm)	1.28±0.96	2.69±1.73	*p*<0.05

**Table 8:** Postoperative adverse events of endoscopic and surgical operation.

postoperative adverse events		endoscopic operation	surgical operation	*p* value
delayed bleeding (N)	Y	1	1	*p*=0.259
	N	142	22	
local infection (N)	Y	3	6	*p*<0.05
	N	140	17	
pneumomediastinum (N)	Y	6	0	*p*=0.597
	N	137	23	
pneumothorax (N)	Y	3	2	*p*=0.142
	N	140	21	
pleural effusion (N)	Y	5	23	*p*<0.05
	N	138	0	
short-term adverse events	Y	10	23	*p*<0.05
	N	133	0	

## References

[1] Seremetis MG, Lyons WS, DeGuzman VC, Peabody JW (1976). Leiomyomata of the esophagus. An analysis of 838 cases. Cancer.

[2] Punpale A, Rangole A, Bhambhani N, Karimundackal G, Desai N, de Souza A (2007). Leiomyoma of esophagus. Ann Thorac Cardiovasc Surg.

[3] Loviscek LF, Yun JH, Park YS, Chiari A, Grillo C, Cenoz MC (2009). [Leiomyoma of the oesophagus]. Cir Esp.

[4] Gupta V, Lal A, Sinha SK, Nada R, Gupta NM (2009). Leiomyomatosis of the esophagus: experience over a decade. J Gastrointest Surg.

[5] Wang L, Ren W, Zhang Z, Yu J, Li Y, Song Y (2013). Retrospective study of endoscopic submucosal tunnel dissection (ESTD) for surgical resection of esophageal leiomyoma. Surg Endosc.

[6] Mutrie CJ, Donahue DM, Wain JC, Wright CD, Gaissert HA, Grillo HC (2005). Esophageal leiomyoma: a 40-year experience. Ann Thorac Surg.

[7] Jiang W, Rice TW, Goldblum JR (2013). Esophageal leiomyoma: experience from a single institution. Dis Esophagus.

[8] Lee LS, Singhal S, Brinster CJ, Marshall B, Kochman ML, Kaiser LR (2004). Current management of esophageal leiomyoma. J Am Coll Surg.

[9] Choong CK, Meyers BF (2003). Benign esophageal tumors: introduction, incidence, classification, and clinical features. Semin Thorac Cardiovasc Surg.

[10] Puli SR, Reddy JB, Bechtold ML, Antillon D, Ibdah JA, Antillon MR (2008). Staging accuracy of esophageal cancer by endoscopic ultrasound: a meta-analysis and systematic review. World J Gastroenterol.

[11] Bonavina L, Segalin A, Rosati R, Pavanello M, Peracchia A (1995). Surgical therapy of esophageal leiomyoma. J Am Coll Surg.

[12] Choi SH, Kim YT, Han KN, Ra YJ, Kang CH, Sung SW (2011). Surgical management of the esophageal leiomyoma: lessons from a retrospective review. Dis Esophagus.

[13] Bardini R, Segalin A, Ruol A, Pavanello M, Peracchia A (1992). Videothoracoscopic enucleation of esophageal leiomyoma. Ann Thorac Surg.

[14] Izumi Y, Inoue H, Endo M (1996). Combined endoluminal-intracavitary thoracoscopic enucleation of leiomyoma of the esophagus. A new method. Surg Endosc.

[15] Kabuto T, Taniguchi K, Iwanaga T, Terasawa T, Tateishi R, Taniguchi H (1980). Diffuse leiomyomatosis of the esophagus. Dig Dis Sci.

[16] Xu GQ, Qian JJ, Chen MH, Ren GP, Chen HT (2012). Endoscopic ultrasonography for the diagnosis and selecting treatment of esophageal leiomyoma. J Gastroenterol Hepatol.

[17] Lu J, Jiao T, Zheng M, Lu X (2014). Endoscopic resection of submucosal tumors in muscularis propria: the choice between direct excavation and tunneling resection. Surg Endosc.

